# Draft Genome Sequence of a Leptospira kirschneri Serovar Mozdok Type 2 Strain Isolated from a Horse in Portugal

**DOI:** 10.1128/MRA.00217-21

**Published:** 2021-07-15

**Authors:** João Inácio, Antonio Muñoz-Mérida, Ahmed A. Ahmed, Teresa Rocha, João Rodrigo Mesquita, Gertrude Thompson, Marga G. A. Goris, Rudy A. Hartskeerl, Ana Sofia Ferreira

**Affiliations:** a Unidade de Microbiologia Médica, Global Health and Tropical Medicine, Instituto de Higiene e Medicina Tropical, Universidade Nova de Lisboa, Lisbon, Portugal; b School of Pharmacy and Biomolecular Sciences, University of Brighton, Brighton, United Kingdom; c Research Center in Biodiversity and Genetic Resources, University of Porto, Vila do Conde, Portugal; d OIE and National Collaborating Centre for Reference and Research on Leptospirosis, Academic Medical Center, Department of Medical Microbiology, University of Amsterdam, Amsterdam, the Netherlands; e Instituto Nacional de Investigação Agrária e Veterinária, I.P., Unidade Estratégica de Produção e Saúde Animal, Oeiras, Portugal; f Epidemiology Research Unit, Instituto de Saúde Pública da Universidade do Porto, Porto, Portugal; g Institute of Biomedical Sciences Abel Salazar, University of Porto, Porto, Portugal; h Laboratory of Microbiology, Department of Biological Sciences, Faculty of Pharmacy, University of Porto, Porto, Portugal; University of Maryland School of Medicine

## Abstract

Leptospira kirschneri is an agent causing leptospirosis in animals and humans. We report the draft genome sequence of Leptospira kirschneri serovar Mozdok type 2 strain Horse 112, comprising 485 contigs and having a genome size of 4,301,784 bp. This genome will facilitate studying important mechanisms for clinical outcomes.

## ANNOUNCEMENT

Leptospirosis is a worldwide underestimated zoonotic disease that occurs in animals and humans and presents a wide variety of symptoms, ranging from very mild febrile illness to life-threatening complications (1, 2). Leptospira kirschneri is a pathogenic species, and the Mozdok serovar, within the Pomona serogroup, is associated with leptospirosis in animals and also in humans (3–6). Mozdok strains comprise three types (1, 2, and 3) based on the different patterns obtained from the agglutination of a panel of monoclonal antibodies (7). However, very little is known regarding the genetic differentiation of these three Mozdok serovar types, and to date no genome sequence of a serovar Mozdok type 2 strain is publicly available. Here, we announce the draft genome sequence of Leptospira kirschneri serovar Mozdok type 2 strain Horse 112, which was isolated from a horse in Portugal (8). The strain was obtained during an abattoir survey performed in Portugal in 2002 and 2003 by the leptospirosis group of the Instituto Nacional de Investigação Agrária e Veterinária (INIAV); it was originally typed as serovar Tsaratsovo by restriction endonuclease analysis (REA) (8) but was later retyped as Mozdok type 2 (6).

Genomic DNA was extracted using the QIAamp DNA extraction kit (Qiagen, Hilden, Germany), following the manufacturer’s instructions with slight modifications. Briefly, leptospires were grown in liquid Ellinghausen-McCullough-Johnson-Harris (EMJH) medium at 28°C, and DNA was extracted from 25 ml of pelleted leptospires (45 min at 16,000 × *g*) in EMJH medium resuspended in ATL buffer.

High-throughput sequence data were generated using an Illumina HiSeq 2500 sequencer with paired-end reads for 100× coverage. A total of 2,872,378 reads were produced, with a mean length of 295 bp. Adapter-trimmed high-quality reads were assembled using the CLC Genomics Workbench v7.0.3 *de novo* assembler. The assembly contains 485 contigs, which encompass 4,301,784 bp (*N*_50_ value of 15,462 bp), while the GC content is 35.9%. The assembly quality was evaluated through Benchmarking Universal Single‐Copy Orthologs (BUSCO) v3.1.0 using the spirochete database, which returned 215 complete BUSCOs (90.7%), 17 fragmented BUSCOs (7.2%), and only 5 missing BUSCOs (2.1%) of the 237 BUSCOs tested.

Genome annotation was performed using Prodigal v2.6.3 for coding regions with parameters –f gff –a and Infernal v1.1.2 for noncoding regions, limiting the search to tRNAs and rRNAs, through the cmscan algorithm with parameters --noali --tblout. A total of 3,766 genes were detected, as well as 38 tRNAs (one of them truncated at the 5′ end), one 5S rRNA gene, seven 23S rRNA genes, and one 16S rRNA gene.

Coding proteins coming from the gene predictions with Prodigal were functionally annotated with the software Sma3s v2. Of the 3,766 proteins, 3,109 had functional annotation and 2,943 included a gene name identified through an orthologous sequence in Uniref90. Gene Ontology (GO) terms from three categories (cellular component, molecular function, and biological process) were associated with the proteins, together with enzyme codes, pathways, and keywords from UniProt. The description of the draft genome sequence of a Leptospira kirschneri serovar Mozdok type 2 strain, given the scarcity of information about these strains worldwide, provides new tools to aid the identification of potentially important pathogenicity mechanisms in these agents.

### Data availability.

The draft genome sequence of Leptospira kirschneri serovar Mozdok type 2 strain Horse 112 was deposited in DDBJ/ENA/GenBank under the accession number WIQJ00000000. Raw data (SRA accession number SRR10301832) were also submitted under BioProject PRJNA578040.
